# Estimating the Healthiness of Internet Recipes: A Cross-sectional Study

**DOI:** 10.3389/fpubh.2017.00016

**Published:** 2017-02-13

**Authors:** Christoph Trattner, David Elsweiler, Simon Howard

**Affiliations:** ^1^Department of New Media Technology, MODUL University Vienna, Vienna, Austria; ^2^Fakultät für Sprach-, Literatur- und Kulturwissenschaften, University of Regensburg, Regensburg, Germany; ^3^Northumbria University, Coach Lane Campus (East), Newcastle upon Tyne, UK

**Keywords:** nutrition, internet recipes, obesity, food, public health

## Abstract

A government’s response to increasing incidence of lifestyle-related illnesses, such as obesity, has been to encourage people to cook for themselves. The healthiness of home cooking will, nevertheless, depend on what people cook and how they cook it. In this article, one common source of cooking inspiration—Internet-sourced recipes—is investigated in depth. The energy and macronutrient content of 5,237 main meal recipes from the food website Allrecipes.com are compared with those of 100 main meal recipes from five bestselling cookery books from popular celebrity chefs and 100 ready meals from the three leading UK supermarkets. The comparison is made using nutritional guidelines published by the World Health Organization and the UK Food Standards Agency. The main conclusions drawn from our analyses are that Internet recipes sourced from Allrecipes.com are less healthy than TV chef recipes and ready meals from leading UK supermarkets. Only 6 out of 5,237 Internet recipes fully complied with the WHO recommendations. Internet recipes were more likely to meet the WHO guidelines for protein than other classes of meal (10.88 v 7% (TV), p < 0.01; 10.86 v 9% (ready), p < 0.01). However, the Internet recipes were less likely to meet the criteria for fat (14.28 v 24 (TV) v 37% (ready); p < 0.01), saturated fat (25.05 v 33 (TV) v 34% (ready); p < 0.01), and fiber (compared to ready meals 16.50 v 56%; p < 0.01). More Internet recipes met the criteria for sodium density than ready meals (19.63 v 4%; p < 0.01), but fewer than the TV chef meals (19.32 v 36%; p < 0.01). For sugar, no differences between Internet recipes and TV chef recipes were observed (81.1 v 81% (TV); p = 0.86), although Internet recipes were less likely to meet the sugar criteria than ready meals (81.1 v 83% (ready); p < 0.01). Repeating the analyses for each year of available data shows that the results are very stable over time.

## 1 Introduction

Lifestyle-related illnesses, such as diabetes and obesity, have high social and economic costs. Globally, more than two-thirds (68.8%) of adults and almost 3 in 4 men (74%) are considered to be overweight or obese (1). In 2008, the costs related to obesity in the US alone were estimated to be $147 billion (2). The situation with respect to diabetes is no better. In 2015, 415 million people were estimated to have diabetes worldwide (1 in 11 adults), and the annual costs are estimated to be $673 billion (12% of global health expenditure) (3).

One of the major contributing factors to such illnesses is poor dietary habits, in particular diets high in sugar, carbohydrates, and fat and diets low in fiber (4–6). Considerable research attention and practical intervention measures have been taken in an attempt to improve dietary choices. One such measure has been to encourage people to cook for themselves at home. For example, both the US Government’s ChooseMyPlate initiative (7) and the UK Government’s Change4Life program (8) advocate home cooking. Poorer cooking skills, less-frequent preparation of home-cooked food, and more frequent consumption of pre-prepared foods have been associated with restricted quality of diet and obesity (9–11). Further evidence supporting the endorsement of home cooking comes from research demonstrating that the amount of food consumed away from home is linked with higher Body Mass Index and lower fruit and vegetable intake (12), leading the authors of one study to conclude that strategies are needed to encourage more cooking among the general population (12). The healthiness of home cooking will, nevertheless, depend on what people cook and how they cook it. These two variables correlated with sociodemographic factors and obesity (13). Thus, the solution may be more complicated than simply getting people to cook—we need to understand sources of cooking inspiration and the role these play in the dietary decisions people make.

Howard et al. (14) investigated the nutritional properties of two common sources of food. They compared recipes published by well-known UK celebrity chefs with leading UK supermarket ready meals, which are often presented as being unhealthy (7, 8). Ready meals, more commonly known as “TV dinners” in the United States, are pre-prepared main courses that can be reheated in their container, require no further ingredients, and need only minimal preparation before consumption. Surprisingly, Howard et al. (14) found that in some respects the ready meals were healthier than recipes. More ready meals than recipes met the WHO goals for fiber density and percentage of energy derived from carbohydrate and fat, although more ready meals than recipes exceeded the recommended sodium density. Thus, the source and, in particular, the content of the recipe seem to be more important than the type of meal (i.e., whether it is a ready meal or home cooked).

A further common source of cooking inspiration is the Internet (15). The food website, which, at the time of writing, claims to be the world’s largest food-focused social network, is Allrecipes.com. The site has a community of 40 million home cooks accessing 3 billion pages annually across 19 sites in 24 countries with recipes available in 13 languages (16). The British version of Allrecipes.com was named as the Daily Mail’s top pick for healthy eating websites, highlighting the “sophisticated search engine” and claiming that “… diabetics, coeliacs and even those specifically wanting to increase their fibre intake – are all catered for” (17).

The recommendation of a popular tabloid newspaper combined with government and media encouragement may persuade members of the public that cooking recipes sourced from the Internet is an approach likely to improve their diet, despite this no systematic study having comprehensively assessed the nutritional content of online recipes.

Using methods previously applied to evaluate the healthiness of ready meals and recipes published by celebrity chefs (14), we analyze the energy, protein, carbohydrate, fat, sugar, fiber, and salt content of recipes uploaded to Allrecipes.com and determine whether the nutritional content complied with national and international recommendations. We use the data collected from the previous analysis as a basis of comparison (14).

## 2 Materials and Methods

We carried out a cross-sectional analysis of the nutritional content of 5,237 recipes from the food website Allrecipes.com. The global version of the site was chosen rather than the British version as the British version alone included insufficient data for analysis. This is unlikely to be a significant source of bias as the global version of the site is popular in Britain as well as worldwide: the web analytics service Alexa.com ranks Allrecipes.com as the most popular cooking community platform (18).

The data describing the Allrecipes.com recipes was downloaded in Summer 2015 and contains 7648 recipes published between the years 2000 and 2010 on the Allrecipes.com website as main dishes.

Rather than sampling from the dataset (as was done with the ready meals and celebrity recipes), we used all of the recipes available, which meet the criteria applied in the previously published analysis (14). This was important as we wished to draw comparisons with the meals analyzed in the previous work. The following restrictions were applied:

- we included only main dishes consisting of at least 225 g per portion, matching the restriction used in Howard et al.’s analyses.
- we restricted the recipes to those added before the end of year 2010 as these would have been the recipes available to users of the site at the time of Howard et al.’s analyses.
- we chose only recipes for which reliable nutritional information could be provided (see below).

Table 1 shows how these filters influenced the number of recipes analyzed. For the other categories of meal (TV chef recipes and supermarket ready meals), the data were provided by the lead author of an earlier study (14). Full details of the inclusion and exclusion criteria and sampling methodology for these recipes can be found in their paper (14). Ethical approval was not required for this analysis of published, publicly available information.

**Table 1:** **Basic statistics of the Internet recipes dataset obtained from Allrecipes.com**.

	Number of recipes	Percentage of recipes
Total published main dish recipes	7,648	100
Contains nutrition information	7,619	99.62
Has at least 225 g per serve	5,237	68.47

### 2.1 Nutritional Content of Included Internet Recipes

The following nutritional information is available in our dataset about each recipe: year of publication; the recommended number of servings; and total energy (kJ), protein (g), carbohydrate (g), sugar (g), sodium (mg), fat (g), saturated fat (g), and fiber (mg) content.

The nutritional metadata was available *via* Allrecipes.com and collected during the main crawl. Allrecipes.com estimates the nutritional content for an uploaded recipe by matching the contained ingredients with those of the ESHA research database (19).

A small number of the main dish recipes (29 in total) collected have no nutritional information available. These recipes were excluded from our analyses. How these exclusion criteria influenced the number of recipes analyzed is shown in Table 1.

### 2.2 Statistical Analysis

Throughout our analyses, we make use of two internationally recognized standards for measuring the healthiness of meals: the World Health Organization (WHO) guidelines (20) and the UK FSA “traffic light” system for labeling food (6).

The WHO has defined 15 ranges of macronutrients, which should be considered in a daily meal plan. We follow the approach of Howard et al. (14) who chose the 7 most important (i.e., proteins, carbohydrates, sugars, sodium, fats, saturated fats, and fibers) and their corresponding ranges to determine a so-called WHO health score. The scale ranges from 0 to 7 (0 meaning none of the WHO ranges are fulfilled and 7 meaning all ranges are met). A recipe or meal plan with a WHO score of 7 is interpreted as being very healthy, whereas a score of 0 is seen as very unhealthy.

A similar approach is taken to derive an FSA traffic light labeling system score. The FSA score relates only to 4 macronutrients (sugar, sodium, fat, and saturated fat). The scale is green (healthy), amber, and red (unhealthy).

Following the procedure described in Howard et al.’s paper (14), for each meal, we calculated the nutritional content per portion by dividing the total content by the number of portions in the meal. Using the Mann–Whitney test, we compared the total content per portion between the Internet recipes and ready meals, as well as Internet recipes and recipes from TV chefs.

We calculated the percentage of energy derived from each macronutrient for each meal and used the Mann–Whitney test to compare the differences between the groups of ready meals and recipes, as well as recipes from the Internet and TV chefs. Using *χ*^2^ tests, we compared the percentage of energy derived from macronutrients in the meals of different types with the nutrient intake goals for preventing diet-related chronic diseases recommended by WHO (6).

For each meal in different groups, we assigned a “traffic light” color for the four macronutrients (fat, saturated fat, sugar, and salt) according to a modified version of the 2007 FSA guidance on its recommended labeling scheme (20). Our modification was that, due to the fact that data on the proportion of sugar derived from such sources were not available, we did not include the criterion allowing a higher total sugar content in situations, where a high proportion of sugar is derived from natural sources. This mirrors the procedure applied in Howard et al.’s work (14). The traffic light system is used on the front of packaging to help consumers assess at a glance the fat, saturated fat, sugar, and salt content of meals, with the aim of helping them to make healthier dietary choices.

In addition to the statistical analyses performed by Howard et al., we calculated the same values for recipes published each year on Allrecipes.com. This allowed us to determine how stable the results are over time.

## 3 Results

Table 2 shows the nutritional content per portion of Internet recipes created by users in Allrecipes.com compared to TV chef recipes and supermarket ready meals. The TV chef recipes contain more energy (2,530 v 2,113 kJ (Internet) v 2,066 kJ (ready)) and more protein (37.46 v 29.50 g (Internet) v 27.85 g (ready)) than both Internet recipes and ready meals (all comparisons sig. p < 0.01). Internet recipes are significantly lower in terms of carbohydrate (35.20 v 49.48 g (TV) v 51.05 g (ready), p < 0.01) and sugar (5.50 v 8.25 g (TV) v 6.80 g (ready), p < 0.01, p = 0.02) content, but significantly higher than TV chef recipes in terms of sodium density (829 v 660 mg, p < 0.01). There is no significant difference between the sodium content of Internet recipes and ready meals, which are known for unhealthy high levels of salt (21, 22) (829 v 800 mg, p = 0.59). In terms of fats and saturated fats, recipes from TV chefs contain the most (fat: 27.06 g, saturated fat: 9.20 g), but ready meals contain the least (fat: 17.20 g, saturated fat: 6.80 g) the difference between ready meals and Internet recipes being highly significant (fat: 17.20 v 24.80 g, p < 0.01; saturated fat: 6.80 v 8.80 g, p < 0.01). The fiber content of Internet recipes is low in comparison to the meals from the other sources (significantly lower than both TV chef meals 3.00 vs 3.44 g, p = 0.01 and ready meals 6.45 g, p < 0.01).

**Table 2:** **Nutritional content per portion of Internet recipes created by users in Allrecipes.com compared to TV chef recipes and supermarket ready meals as of December 2010**.

Nutritional content	Median (interquartile range)				
	Internet recipesN = 5,237	TV chef recipesN = 100	Ready mealsN = 100	*P* valuea	*P* valueb
Energy (kJ)	2,112.92 (1,598.29–2,723.78)	2,530.27 (2,024.18–3,256.72)	2,066.90 (1,715.44–2,575.25)	<0.01	0.41
Protein (g)	29.5 (2.90–39.20)	37.46 (26.47–50.13)	27.85 (23.18–33.13)	<0.01	0.03
Carbohydrate (g)	35.2 (17.30–53.85)	49.48 (23.48–68.16)	51.05 (41.90–67.40)	<0.01	<0.01
Sugar (g)	5.50 (2.85–9.90)	8.25 (4.86–12.98)	6.80 (4.13–11.10)	<0.01	0.02
Sodium (mg)	829.00 (487.50–1,264.00)	660.00 (365.00–1,042.50)	800.00 (600.00–1,000.00)	0.01	0.59
Fat (g)	24.80 (15.70–36.40)	27.06 (16.80–40.53)	17.20 (12.03–23.90)	0.05	<0.01
Saturated fat (g)	8.80 (4.70–14.20)	9.20 (4.93–16.11)	6.80 (3.65–11.67)	0.26	<0.01
Fiber (g)	3.00 (1.50–5.00)	3.44 (2.21–6.06)	6.45 (4.80–8.70)	0.01	<0.01

*^a^Mann–Whitney test comparing Internet recipes with TV chef recipes*.

*^b^Mann–Whitney test comparing Internet recipes with ready meals*.

**Figure 1:**
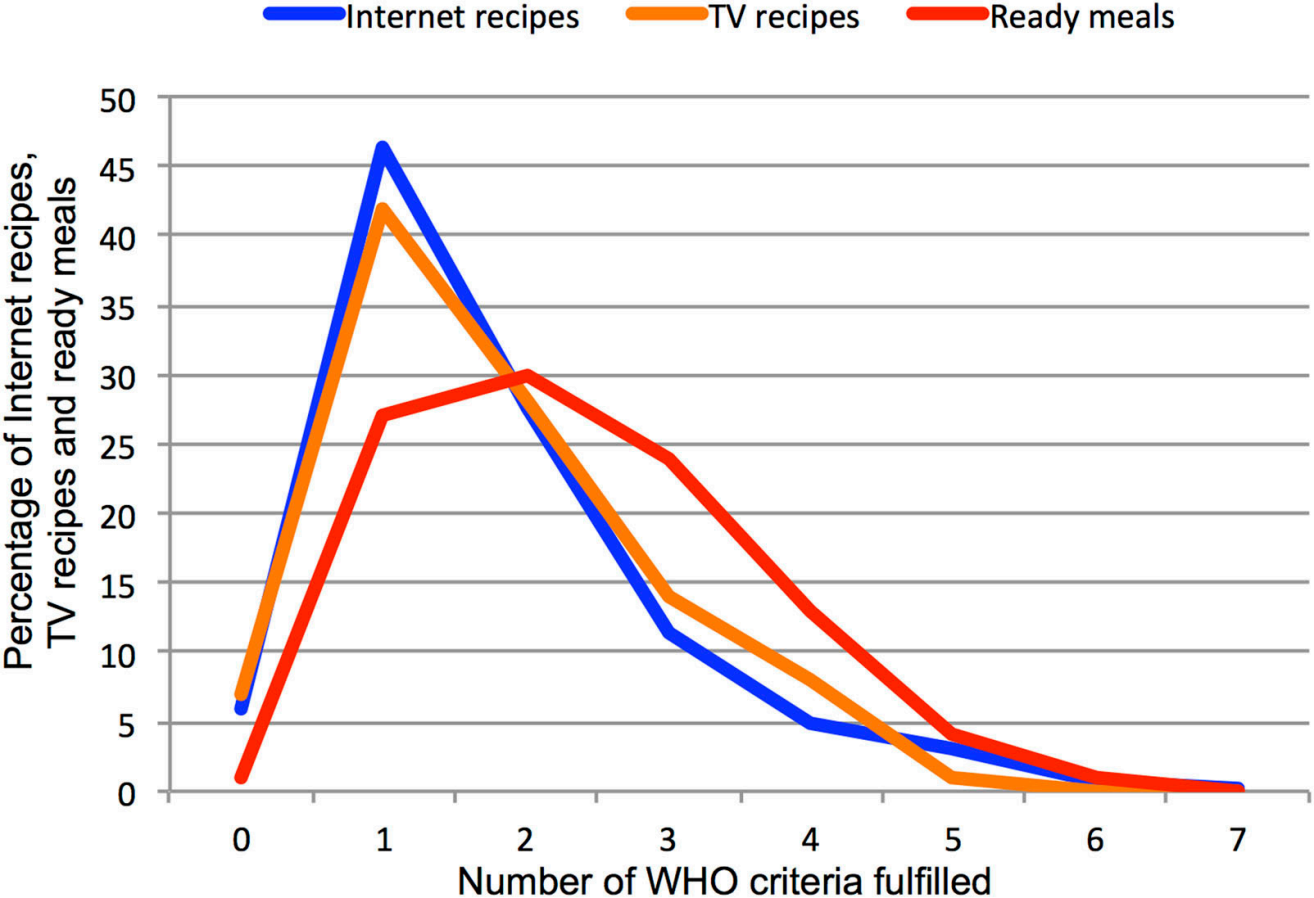
**Comparison of distributions for Internet recipes, TV chef recipes, and ready meals for number of WHO criteria fulfilled**.

Table 3 and Figure 1 summarize the percentage of recipes/meals of different types, which meet the criteria established by the WHO in terms of the individual nutritional properties. In this case, the values are normalized with respect to the total number of calories in one portion. The data show that only 6 of the 5,237 Internet recipes (0.11%) and none of the sampled TV chef recipes or ready meals meet all 7 criteria. A small number of recipes of each type (5.9% of Internet recipes, 7% of TV chef recipes, and 1% of ready meals) do not meet any of the criteria at all. However, the majority recipes of all three types meet one or two criteria. A total of 46% of Internet recipes meet one criterion compared to 42% of TV chef recipes and 27% of ready meals. As the number of criteria to be met increases, the percentage of Internet recipes meeting these drops at a higher rate than the other types of meal. Figure 1 shows that Internet recipes and TV chef recipes met a similar mean number of WHO criteria (1.76 and 1.77, respectively; SDs 1.17 and 1.10, respectively). Ready meals met a mean of 2.37 criteria (SD 1.21). Using Mann–Whitney tests, the small difference between Internet recipes and TV chef recipes is non-significant (p = 0.64), but ready meals met significantly more WHO criteria than Internet recipes (p < 0.01).

**Table 3:** **Comparison of distributions for Internet recipes, TV chef recipes, and ready meals for number of WHO criteria fulfilled as of December 2010**.

Number of WHO criteria fulfilled	Percentage (total)		
	Internet recipesN = 5,237	TV chef recipesN = 100	Ready mealsN = 100
0	5.94 (311)	7	1
1	46.27 (2,423)	42	27
2	27.63 (1,447)	28	30
3	11.34 (594)	14	24
4	4.98 (261)	8	13
5	3.04 (159)	1	4
6	0.69 (36)	0	1
7	0.11 (6)	0	0

Table 4 shows the number and proportion of each meal type that met each nutrient-specific WHO goal. More Internet recipes meet the WHO guidelines for protein than other classes of meal (10.88 v 7% (TV), p < 0.01, 10.86 v 9% (ready), p < 0.01). However, fewer Internet recipes met the criteria for fat (14.28 v 24 (TV) v 37% (ready), p < 0.01), saturated fat (25.05 v 33 (TV) v 34% (ready); p < 0.01), and fiber (when compared to ready meals 16.50 v 56%; p < 0.01). In terms of salt, significantly more Internet recipes met the criteria for sodium density than ready meals (19.63 v 4%, p < 0.01), but significantly fewer than the TV chef meals (19.32 v 36%, p < 0.01). For sugar, no differences between Internet recipes and TV recipes can be observed (81.1 v 81% (TV), p = 0.86). However, Internet recipes significantly differ from ready meals (81.1 v 83% (ready), p < 0.01).

**Table 4:** **Median percentage of energy derived from macronutrients, and sodium and fiber density, of 5,237 Internet recipes, 100 TV chef recipes and 100 supermarket ready meals as of December 2010**.

Nutritional content	Internet recipes (***N*** = 5,237)		TV recipes (***N*** = 100)		Ready meals (***N*** = 100)						
Macronutrient (% energy)	Median (interquartile range)	% within WHO range	Median (interquartile range)	% within WHO range	Median (interquartile range)	% within WHO range	WHO range	*χ*^2^^a^	P value^a^	*χ*^2^^b^	P value^b^
Protein	23.23 (17.94–30.13)	10.88	23.8 (18.8–33.9)	7	22.7 (18.2–27.3)	9	10–15	121.36	<0.01	22.70	<0.01
Carbohydrate	28.38 (15.99–40.97)	7.85	31.6 (19.0–42.1)	6	42.9 (37.0–52.5)	18	55–75	31.71	<0.01	365.68	<0.01
Sugar	4.44 (2.20–8.20)	81.10	5.3 (3.3–8.8)	81	5.7 (3.8–8.7)	83	<10	0.03	0.86	13.45	<0.01
Fat	45.42 (34.53–55.69)	14.28	42.2 (30.1–54.0)	24	32.4 (25.9–39.2)	37	15–30	271.10	<0.01	1,159.43	<0.01
Saturated fat	15.91 (9.97–22.01)	25.05	14.9 (9.0–20.9)	33	13.9 (7.8–18.7)	34	<10	149.61	<0.01	186.84	<0.01
Fiber density (g/MJ)	1.41 (0.72–2.41)	16.80	1.4 (0.8–2.6)	14	3.2 (2.4–4.4)	56	>3.0	34.19	<0.01	3,265.40	<0.01
Sodium density (g/MJ)	0.40 (0.24–0.60)	19.63	0.2 (0.1–0.4)	36	0.4 (0.3–0.5)	4	<0.2	609.15	<0.01	3,331.54	<0.01

*^a^χ^2^ tests with one degree of freedom comparing proportion of Internet recipes with proportion of TV recipes in World Health Organization range*.

*^b^χ^2^ tests with one degree of freedom comparing proportion of Internet recipes with proportion of ready meals in World Health Organization range*.

*Based on 8.4 MJ/day (2,000 kcal/day) diet and recommended daily fiber intake of >25 g*.

*Based on 8.4 MJ/day (2,000 kcal/day) diet and recommended daily sodium intake of <2 g*.

**Figure 2:**
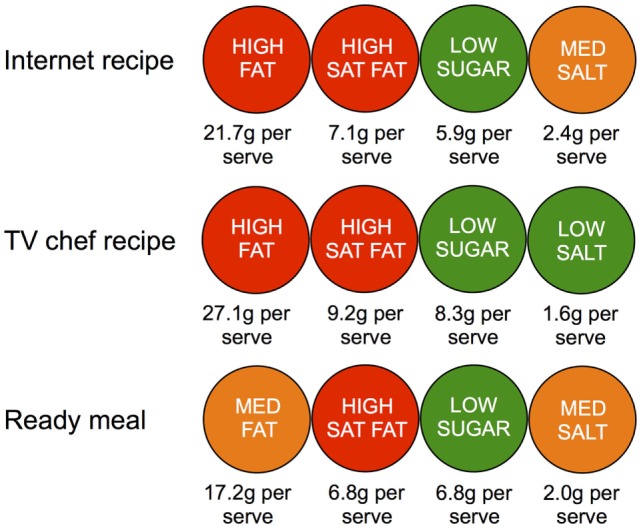
**Simulated front of package labels for an average Internet recipe created by a user in Allrecipes.com, recipe created by a television chef and an own brand supermarket ready meal, based on guidelines from the FSA**.

Table 5 shows the traffic light assessment for the different recipe types according to modified Food Standards Agency guidelines (20). The FSA guidelines are based on macronutrient properties normalized by portion size. According to these guidelines, Internet and TV chef recipes have almost equal numbers of red labels (45 v 47% (TV)), but both TV recipes and ready meals have more green labels than Internet-sourced recipes (36 v 42 (TV) v 41% (ready)). Figure 2 shows these data averaged over all recipes of each type to provide simulated front of package labels for an average Internet recipe, an average TV chef recipe, and an average ready meal using a design based on FSA guidelines. Internet and TV recipes are classified as having high fat; all three categories are labeled as having low sugar, and both ready meals and Internet recipes are considered to contain medium salt.

**Table 5:** **Traffic light assessment according to modified Food Standards Agency guidelines for 5,237 Internet recipes compared to 100 recipes by television chefs and 100 supermarket ready meals as of December 2010**.

FSA label	Internet recipes% within FSA range (total)			TV Recipes% within FSA range (total)			Ready meals% within FSA range (total)		
Macronutrients	Red	Amber	Green	Red	Amber	Green	Red	Amber	Green
Sugar	14 (719)	1 (46)	85 (4,472)	17	0	83	11	0	89
Fat	60 (3,134)	28 (1,443)	13 (660)	68	17	15	37	39	24
Saturated fat	67 (3,490)	7 (374)	26 (1,373)	71	1	28	56	1	43
Salt	42 (2,175)	38 (1,970)	21 (1,092)	31	28	41	30	60	10
Totals	45 (9,518)	18 (3,833)	36 (7,597)	47 (187)	11 (46)	42 (167)	34 (134)	25 (100)	41 (166)

Overall based on the FSA guidelines, the healthiest category is the ready meals and the unhealthiest category is Internet recipes, which is the same overall conclusion drawn when using the WHO guidelines.

**Figure 3:**
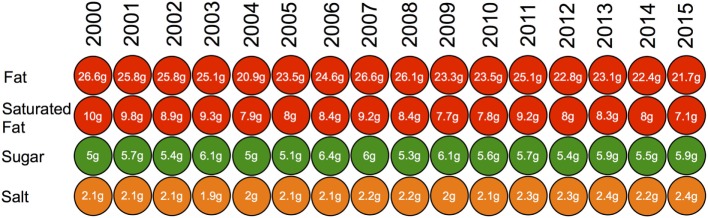
**Simulated front of package labels for an average Internet recipe created by a user in Allrecipes.com based on guidelines from the FSA between the years 2000 and 2015**.

**Figure 4:**
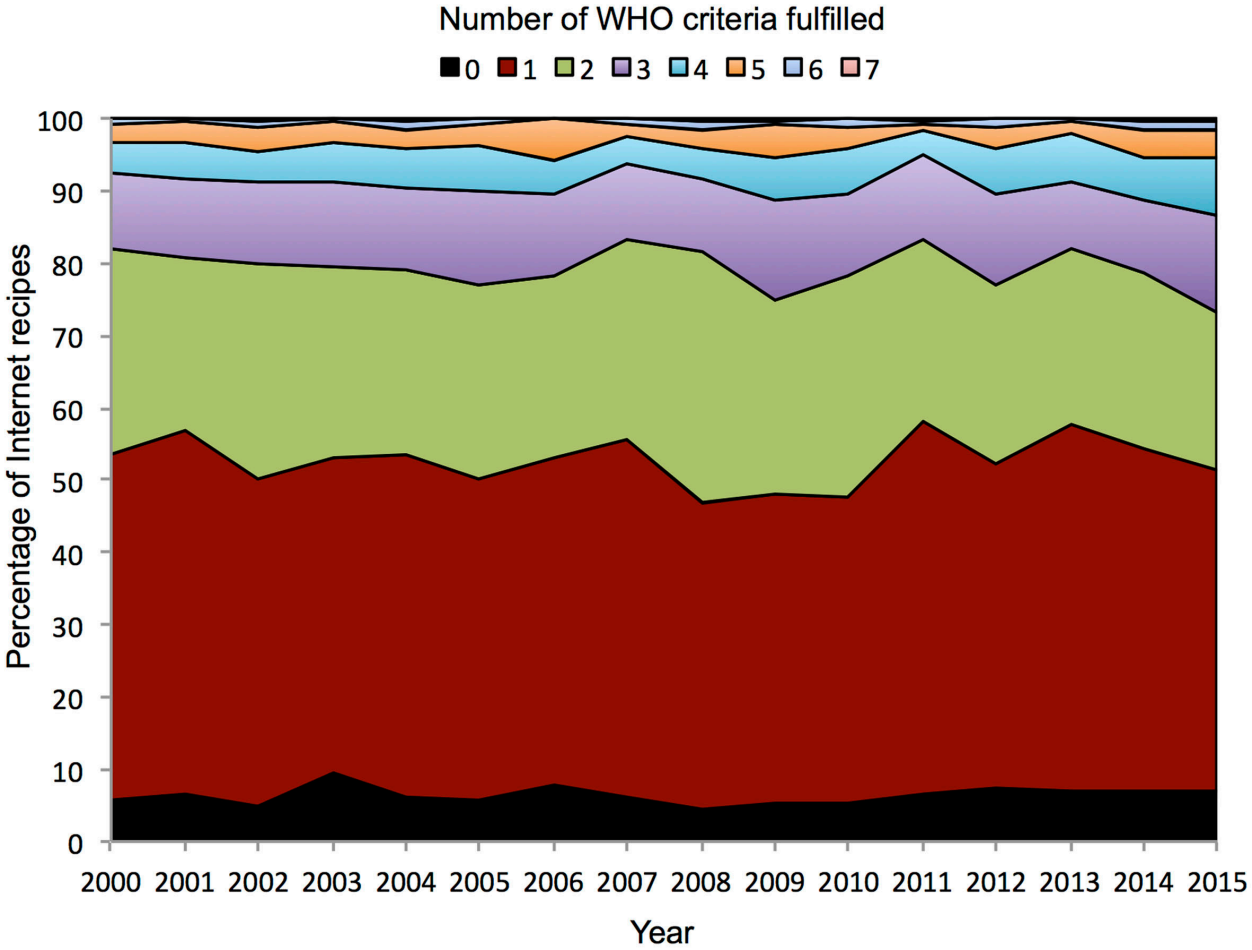
**Percentage of Internet recipes fulfilling the WHO inclusion criteria (from 0 to 7) between the years 2000 and 2015**.

**Figure 5:**
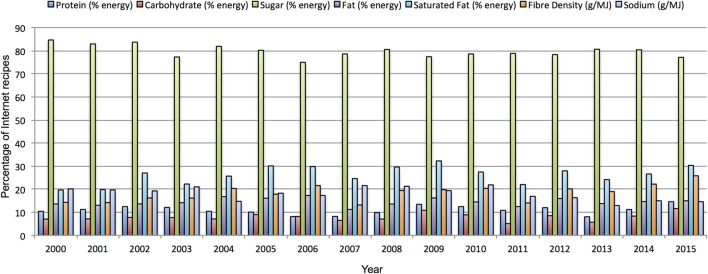
**Percentage of Internet recipes (macronutrition level) fulfilling the WHO inclusion criteria (from 0 to 7) between the years 2000 and 2015**.

To establish how stable these values are over time, we calculated the same statistics for each year of data available to us. Table 6 shows the nutritional content per portion of Internet recipes created by users in Allrecipes.com for the years 2000 to 2015. Similarly, Figure 3 presents the simulated front of package labels, based on the guidelines from the FSA (20), for an average Internet recipe created by Allrecipes.com users for each year during that time period. Overall both depict stable trends over time. The FSA labels for macronutrients, for the average recipe based on annually uploaded recipes, are the same for every year in the dataset. Figure 4 demonstrates the percentage of Allrecipes.com recipes meeting different numbers of WHO criteria overall, and Figure 5 depicts the same information at a macronutrient granularity. Both figures show limited annual variation but present an overall consistent trend.

**Table 6:** **Nutritional content per portion of Internet recipes created by users in Allrecipes.com over time (2000–2015)**.

	Median															
Year Nutritional content	2000N = 1103	2001N = 711	2002N = 425	2003N = 247	2004N = 393	2005N = 465	2006N = 208	2007N = 463	2008N = 324	2009N = 505	2010N = 393	2011N = 313	2012N = 808	2013N = 595	2014N = 526	2015N = 267
Energy (kJ)	2,204.97	2,142.21	2,138.02	2,121.29	1,907.9	2,004.14	2,154.76	2,204.97	2,221.7	2,045.98	2,004.14	2,133.84	2,043.88	2,008.32	1,951.84	1,912.09
Protein (g)	29.8	29.2	28.7	27.7	26.8	30.2	31.9	31.6	33.85	27.6	29.1	30	28.45	28.6	27.9	26.2
Carbohydrate (g)	37.2	36.4	36.5	32.3	32.8	33.9	34.45	34.4	34.65	37.1	34.1	34.3	33.05	35.8	34.6	38.2
Sugar (g)	5	5.7	5.4	6.1	5	5.1	6.35	6	5.3	6.1	5.6	5.7	5.4	5.9	5.5	5.9
Sodium (mg)	829	854	833	769	787	827	843.5	868	876	815	821	900	930	946	872.5	943
Fat (g)	26.6	25.8	25.8	25.1	20.9	23.5	24.55	26.6	26.05	23.3	23.5	25.1	22.8	23.1	22.4	21.7
Saturated Fat (g)	10	9.8	8.9	9.3	7.9	8	8.4	9.2	8.4	7.7	7.8	9.2	8	8.3	7.95	7.1
Fiber (g)	3	3	3.2	2.9	3	2.9	2.85	2.8	2.9	3.1	3.2	2.9	3.2	3.1	3.2	3.7
Size (g)	334.37	330.59	325.82	326.01	319.58	335.26	348.05	346.53	355.52	338.67	344.74	348.06	345.76	342.64	342.11	333.85

## 4 Discussion

Internet recipes sourced from Allrecipes.com tend to be high in protein, fat, saturated fat, and sodium, low in carbohydrate, and within the recommended range for sugar according to World Health Organization’s nutritional guidelines for the avoidance of diet-related diseases (6).

When compared to meals based on television chef recipes and ready meals from leading UK supermarkets, the Internet recipes were the least healthy. Significantly, fewer were within the recommended ranges for fat, saturated fat compared to meals from the other sources, and significantly fewer Internet recipes met the recommended range for fiber than ready meals. In terms of sodium density, significantly more Internet recipes met the criteria than ready meals, but significantly fewer than the TV chef meals. Internet recipes did, however, meet the criteria for protein content significantly more often than the other categories.

Internet recipes were also more likely to achieve red traffic light labels according to the criteria of the UK Food Standards Agency (FSA) (20). By investigating the criteria for each year of data collection, we revealed that the characteristics of recipes uploaded to the site are very stable on average. Thus, our findings, taken together with those of Howard et al., add weight to the argument that encouraging people to cook more at home might not, by itself, be enough to improve nutritional habits. This is because some common sources of cooking inspiration themselves promote unhealthy meals.

### 4.1 Strengths and Weaknesses of the Study

This study is the first to comprehensively evaluate the nutritional content of Internet-sourced main meals. To our knowledge, it is not only the largest study investigating the healthiness of online sourced recipes but also the first to systematically compare the nutritional properties of Internet recipes with those of other sources of cooking inspiration. Moreover, it is also the first investigation to provide insights on the nutritional content of Internet recipes over a long period of time (16 years in total). Although we examined the food website with the largest traffic volumes on the Internet, it is possible (and perhaps likely) that recipes from different Internet sources may have provided different results.

A second limitation of the study is that we have analyzed the Allrecipes.com collection as a whole. The collection is not subject to the same editorial or space constraints as a book or supermarket shelf, and thus no restrictions are placed on the recipes published on the site. It could be, therefore, that certain classes of recipes (e.g., Vegetarian, Asian, Italian, gluten-free, etc.) are over or underrepresented in the collection, which would bias the findings. Moreover, it could be that recipes, popular with users of the food portal, have different nutritional properties to unpopular recipes. Further analyses we performed confirm this (23). We now know that, for example, recipes accepted by users (i.e., recipes bookmarked most often and rated most highly) tend to be on average the least healthy. We also know that recipes associated with particular categories on the site are healthier than others according to the health guidelines we applied and users are not adept at distinguishing, which categories these might be. Although the new analyses were not performed with respect to comparing with other sources of meals as we have done here, they nevertheless endorse and strengthen the conclusions we have drawn based on the findings reported in this paper.

A further limitation of the study is that Allrecipes.com is an international site and a large percentage of the recipes are sourced from the US, whereas the TV chef recipes and ready meals were sourced from the UK. However, access statistics show that the site is among the most popular food sites in the UK, although the UK localized version (which was not available for the full duration of the period for which we wanted to draw comparison) has since overtaken the international version in terms of numbers of visits from UK users.

The datasets describing the supermarket ready meals and cookery books were created sometime ago and published in Howard et al.’s work (14) in 2012. All of the ready meals and books tested are still available for purchase at the time of writing.

It is important to acknowledge that the nutritional information for the three groups of meals was collated using different methods—for ready meals, the manufacturer’s data was taken; for Internet recipes, Allrecipes.com data was taken; the TV chef recipes were analyzed using the WinDiets software (24). However, we would expect, based on the comparisons already reported in the medical literature (14), these methods to have broadly comparable results.

To evaluate the health properties of recipe and meals, we used metrics based on guidelines from the World Health Organization and the UK Food Standards Agency. These choices were driven by precedence in the literature (14). It could be argued that the WHO score might not be the best measure to determine the healthiness of an individual recipe or meal because it was designed to evaluate whole diet meal plans. Nevertheless, in our opinion, it is still a useful measure as it shows similar health trends to the FSA score but incorporates a wider range of macronutrients in the metric. Our later analyses in Ref. (23) show that there is a strong and significant correlation between the WHO and FSA scores for Allrecipe.com recipes.

Systematic variation from the true macronutrient value of the foods could be a source of bias in this study. In the European Union, which at the time of writing includes the United Kingdom, the published nutritional data used for analysis of ready meals are permitted by law to vary by up to 20% from the true macronutrient values (25). Similarly, establishing the nutritional content of a recipe using ingredient mapping, as is done by Allrecipes.com, is known to be imperfect (26). However, in all cases, our analyses were based on the most accurate data currently available to the public.

We also concede that variation may exist in the way users of Allrecipes.com might use or consume recipes. For example, people may not follow all the steps and ingredients guides in the recipes, which may result in different nutritional intake. Similarly, recipes can be combined, perhaps with a side dish, which may or may not be likely with a ready meal. It is not possible with this kind of analysis to account for such a variation.

As Howard et al. (14) also conceded in their article, our analyses may have systematically mis-estimated the salt content within the recipe groups. Many recipes have the ingredient salt listed with the quantity marked as “a pinch” or “to taste.” Whereas Howard et al. ignored listed salt entries completely, we used the standard values calculated by Allrecipes.com. This website has fixed rules for these quantities, but in practice individual users may apply salt liberally or conservatively based on their own individual tastes. Therefore, the findings for salt, and in particular the comparison with TV chef recipes, should be interpreted with caution.

In our study, individual recipes were analyzed, but a healthy diet is created by combining a variety of food types. Past work has suggested combining recommended online recipes in a manner such that a created daily meal plan meets guidelines from official health organizations (27). This possibility is not reflected in our study.

### 4.2 Comparison with Other Studies

The primary investigation with which we can draw comparison is the study by Howard et al. (14). As reported above, with respect to the WHO and FSA guidelines, the Internet-sourced recipes were evaluated to be less healthy overall.

A 2010 study by Silva et al. (28) applied a healthy recipe index to 204 recipes featured on 2 Food Network shows and found that recipes were ranked as less than healthy by the index measure. Further analysis found that the recipes analyzed were excessive in energy, saturated fat, and sodium based on a 2,300-kcal diet.

While there is a general lack of research on the healthiness of online recipes, one relevant study comes from Schneider and colleagues, who investigated the nutritional properties of recipes (entrees and main dishes) sourced *via* popular online food blogs (29). The dishes were evaluated using US Department of Agriculture and US Department of Health and Human Services, Dietary Guidelines. The recipes analyzed met energy recommendations but were excessive in saturated fat and sodium.

### 4.3 Unanswered Questions and Future Research

When investigating the nutritional content of recipes from food blogs, Schneider et al. (29) found that all risk-related nutrients of interest were significantly lower in vegetarian recipes compared with red meat and poultry recipes. One future research direction would be to investigate if this was the case in Allrecipes.com. Schneider et al. also discovered differences in sodium levels across seasons. A further line of research could similarly investigate temporal patterns in the nutritional properties of uploaded recipes.

It would, furthermore, be possible to repeat our analyses for other popular food sites, which can be crawled in a similar manner. Previous work has identified cultural differences in cooking habits *via* online recipe databases (30), and it would be interesting to investigate such cultural differences from the perspective of healthy nutrition.

### 4.4 Meaning of the Study and Implications

The Internet is a technology which the evidence suggests appeals to people as a source of cooking inspiration. Our analyses show that the content provided may not be healthy. As Howard et al. (31) suggest, the home cooking of nutritionally balanced recipes using primarily raw ingredients would likely be a nutritionally superior strategy relying on Internet recipes, recipes by television chefs, or ready meals.

Nevertheless, a problem associated with a technology may have a technological solution. For example, recommendation systems—such as those commonly used in online shops such as http://Amazon.com—have the potential to be used to find recipes of a similar type, but with different nutritional properties (31). Initial steps in this direction have been taken specifically for patients with diabetes (32).

## Author Contributions

CT collected and analyzed the data. DE and CT drafted the manuscript, and both authors contributed to the study design and interpretation of results. SH commented on successive drafts of the manuscript and supported CT with the study design. CT and DE will act as guarantors.

## Conflict of Interest Statement

All authors have completed the ICMJE uniform disclosure form at http://icmje.org/downloads/coi_disclosure.zip (available on request from the corresponding author) and declare: no support from any companies for the submitted work; no relationships with any companies that might have an interest in the submitted work in the previous three years; and no non-financial interests that may be relevant to the submitted work.
